# In Patients Undergoing CRS/HIPEC for Colorectal Adenocarcinoma with Peritoneal Metastases, Presence of Ascites on Computed Tomography Imaging is not a Prognostic Marker for Survival

**DOI:** 10.1245/s10434-022-11718-7

**Published:** 2022-04-16

**Authors:** Ibrahim Said, Inge Ubink, Roos S. G. Ewalds, Johanna G. T. Arkesteijn, Henk M. W. Verheul, Johannes H. W. de Wilt, Helena M. Dekker, Andreas J. A. Bremers, Philip R. de Reuver

**Affiliations:** 1 Department of Surgery, Radboudumc, Radboud Institute for Health Sciences, Nijmegen, The Netherlands; 2 Department of Medical Oncology, Radboudumc, Radboud Institute for Health Sciences, Nijmegen, The Netherlands; 3 Department of Medical Imaging, Radboudumc, Radboud University Medical Centre, Nijmegen, The Netherlands

## Abstract

**Background:**

Cytoreductive surgery (CRS) combined with hyperthermic intraperitoneal chemotherapy (HIPEC) is a potentially curative treatment for patients with colorectal peritoneal metastases (CRPM). Patient selection is key to optimizing outcomes after CRS/HIPEC. The aim of this study was to determine the prognostic value of ascites diagnosed on preoperative imaging.

**Methods:**

A prospective database of patients eligible for CRS/HIPEC between 2010 and 2020 was retrospectively analyzed. The presence of ascites, postoperative complications, overall survival (OS), disease-free survival (DFS), and completeness of cytoreduction were assessed. Univariable and multivariable logistic regression was performed to identify independent predictors for outcome.

**Results:**

Of the 235 included patients, 177 (75%) underwent CRS/HIPEC while 58 (25%) were not eligible for CRS/HIPEC. In 42 of the 177 patients (24%) who underwent CRS/HIPEC, ascites was present on preoperative computed tomography (CT) imaging. Peritoneal Cancer Index (PCI) score was significantly higher in patients with preoperative ascites compared with patients without (11 [range 2–30] vs. 9 [range 0–28], respectively; *p* = 0.011) and complete cytoreduction was more often achieved in patients without ascites (96.3% vs. 85.7%; *p* = 0.007). There was no significant difference in median DFS and OS after CRS/HIPEC between patients with and without ascites {10 months (95% confidence interval [CI] 7.1–12.9) vs. 9 months (95% CI 7.2–10.8), and 25 months (95% 9.4–40.6) vs. 27 months (95% CI 22.4–31.6), respectively}.

**Conclusions:**

Ascites on preoperative imaging was not associated with worse survival in CRS/HIPEC patients with CRPM. Therefore, excluding patients from CRS/HIPEC based merely on the presence of ascites is not advisable.

**Supplementary Information:**

The online version contains supplementary material available at 10.1245/s10434-022-11718-7.

The peritoneum is the second most common recurrence site in patients with colorectal cancer (CRC), accounting for 25–35% of all recurrences.^1^ Cytoreductive surgery (CRS) combined with hyperthermic intraperitoneal chemotherapy (HIPEC) is a potentially curative treatment^2^ and improves the median overall survival (OS) compared with systemic chemotherapy alone.^3^

CRS is associated with severe morbidity and even mortality, which underlines the importance of identifying patients who are most likely to benefit from CRS/HIPEC.^4^ Various research groups have identified prognostic factors for recurrence and survival after CRS/HIPEC for colorectal peritoneal metastases (CRPM), including the Peritoneal Cancer Index (PCI), tumor stage, differentiation grade, and completeness of cytoreduction (CC).^5,6^ These factors are only suitable for postoperative prognostication, while improved patient selection is indicated prior to surgery.

Ascites on preoperative imaging has been suggested as a marker for extent of peritoneal disease. In patients with primary peritoneal mesothelioma and gastric or ovarian tumors, ascites on preoperative imaging is associated with incomplete cytoreduction and poor survival.^7^ Randle et al.^8^ reported on a series of more than 1000 CRS/HIPEC procedures for various primary intestinal tumors and found that complete cytoreduction was obtained in 15% of patients with ascites compared with 59% of patients without. However, the value of ascites as a prognostic factor for survival after CRS/HIPEC for CRPM has not yet been investigated. We hypothesized that ascites on preoperative imaging is a negative prognostic factor and could therefore aid in preoperative patient selection. The current study aimed to determine the prognostic value of ascites, diagnosed on preoperative imaging, on survival in CRPM patients considered for CRS/HIPEC.

## Methods

### Study Design and Patients

Consecutive patients with CRPM who were considered for CRS/HIPEC between 2010 and 2020 at the Radboud University Medical Center, Nijmegen, The Netherlands, were retrospectively included in this study. Patients with appendiceal neoplasms other than adenocarcinoma and those who underwent second and/or third HIPEC procedures were excluded. Prior to surgery, all patients were discussed in a multidisciplinary team meeting involving surgeons, medical oncologists, radiologists, gastroenterologists, and pathologists. This study was performed in accordance with local medical ethical guidelines and the collection of coded data was approved by the local medical ethics committee.

### Preoperative Assessment of Ascites

The ascites scoring system, used to assess the distribution of ascites in the peritoneal cavity on preoperative computed tomography (CT), was described by Randle et al.^8^ The abdominal cavity was divided into nine regions, identical to those used to calculate the PCI score,^9^ excluding the four regions of the small bowel. The presence of ascites was scored in consecutive regions, with one point given when ascites was present and zero points given when ascites was absent; thus, scores ranged between 0 and 9 for each patient. Assessment of ascites was performed independently by two authors (RE and HD).

### Surgical Procedure, Peritoneal Cancer Index, and Completeness of Cytoreduction Score

CRS/HIPEC was performed as previously described.^10^ The PCI was scored during an explorative laparotomy and was categorized into two groups, with a cut-off point at a PCI score of 7. In our practice, patients with an estimated PCI score above 20 are ineligible for CRS/HIPEC. CC was scored using the CC scoring system as follows: CC0, no evidence of disease after CRS; CC1, tumor nodules <0.25 cm after CRS; and CC2 or higher, tumor nodules >0.25 cm.^11^ After cytoreduction, HIPEC was performed with mitomycin C or oxaliplatin, as described by Elekonawo et al.^12^

### Data Collection and Outcomes

A prospective database was reviewed to assess the data of all patients considered eligible for CRS/HIPEC. Data including clinicopathological characteristics, treatment, surgical procedure, and outcomes were collected during work-up, perioperative care, and follow-up. Follow-up consisted of a biannual contrast-enhanced CT scan of the thorax and abdomen in the first 5 years after CRS/HIPEC, along with measurement of the serum tumor markers carcinoembryonic antigen and carbohydrate antigen (CA) 125 and CA19‐9.

The primary outcome of this study was OS, measured from the date of CRS/HIPEC to the date of death, while the secondary outcomes were disease-free survival (DFS) and postoperative morbidity. DFS was defined as the time from the date of CRS/HIPEC to the date of recurrence of disease or death. Cases were censored at last follow-up and postoperative complications were scored according to the Clavien–Dindo classification system.^13^

### Statistical Analyses

Clinicopathological characteristics and surgical outcomes were analyzed using descriptive statistics. For normally distributed groups, the independent Student’s *t*-test was used to compare means, and the Chi-square or Fisher’s exact tests were used for categorical variables. Survival was measured using the Kaplan–Meier method and patients with and without ascites were compared using the log-rank test. The influence of covariates was determined by Cox proportional hazards analysis for variables with a *p*-value <0.05 in univariate analysis and variables that were considered clinically relevant in the literature. A *p*-value <0.05 was considered statistically significant for all tests. All statistical analyses were performed using IBM SPSS Statistics 25 (IBM Corporation, Armonk, NY, USA).

## Results

### Patient Characteristics

**Fig. 1:**
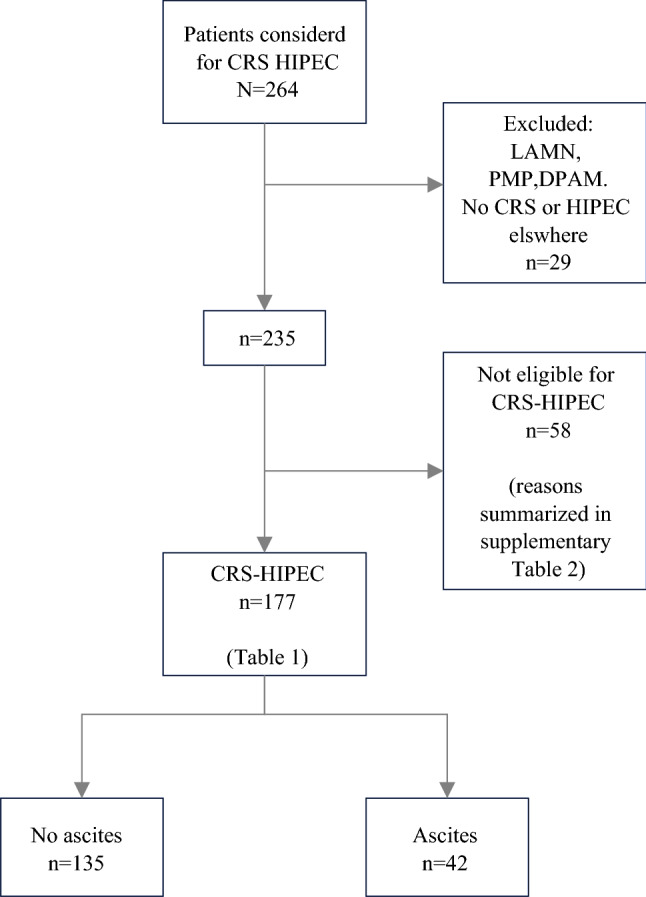
Patient selection process. The flowchart shows the inclusion and exclusion criteria, as well as patients not eligible for CRS/HIPEC. *CRS* cytoreductive surgery, *HIPEC* hyperthermic intraperitoneal metastases, *N* number of patients, *LAMN* low-grade appendiceal mucinous neoplasm, *PMP* pseudomyxoma peritonei, *DPAM* disseminated peritoneal adenomucinosis

A total of 235 patients were included in the present study. Ascites was present on preoperative CT imaging in 58 of 235 patients (25%) who underwent CRS/HIPEC. CRS/HIPEC was performed in 177 patients (75%) and no CRS/HIPEC was performed in 58 patients (Fig. 1). Ascites was present on preoperative CT imaging in 42 of 177 patients (24%) who underwent CRS/HIPEC and in 16 of 58 patients (28%) in the group not eligible for CRS/HIPEC (*p* = 0.203). The ascites distribution for the eligible group was scored as low (1–3 regions) in 26 patients (62%), medium (4–6 regions) in 5 patients (12%), and high (7–9 regions) in 11 patients (26%). Ascites was diagnosed in all regions in 40.5% of patients with ascites (*n* = 17).

Patients who underwent CRS/HIPEC were divided into two groups, based on the presence or absence of ascites (Table 1). These groups were comparable in terms of sex, age, American Society of Anesthesiologists (ASA) classification, onset of peritoneal metastases, and T and N stage of the primary tumor. The PCI score was significantly higher in patients with preoperative ascites compared with those without ascites (11 [range 2–30] vs. 9 [range 0–28]; *p* = 0.011). Complete cytoreduction (CC0) was more common in patients without ascites (96.3% vs. 85.7%; *p* = 0.007). The presence of more ascites, represented by the ascites distribution score (low, medium, and high), was not associated with incomplete cytoreduction (CC1–2). In patients with a low distribution, CC1–2 occurred in 15%; in patients with a medium distribution, CC1–2 occurred in 0%; and in patients with high distribution, CC1–2 occurred in 18% (*p* = 0.944) [electronic supplementary Table 1]. The presence of ascites did not result in higher complication rates or longer hospital stay after CRS/HIPEC (Table 1).

**Table 1:** Baseline characteristics of CRS/HIPEC patients

	All patients	No ascites	Ascites	*p*-Value
	[*N* = 177]	[*n* = 135]	[*n* = 42]	
*Patient characteristics*				
Sex				0.938
Male [n(%)]	81 (45.8)	62 (45.9)	19 (45.2)	
Age, years [mean ± SD]	62.7 ± 11.2	62.9 ± 10.5	62.1 ± 13.3	0.712
ASA				0.137
ASA 3	39 (22.0)	32 (23.7)	7 (16.7)	
Race				
White	177 (100)	135 (100)	42 (100)	
*Preoperative and tumor characteristics*				
pT stage				0.253
4	83 (46.9)	61 (45.2)	22 (52.4)	
pN stage				0.976
1	66 (37.3)	57 (42.2)	9 (21.4)	
2	61 (34.5)	47 (34.8)	14 (33.3)	
Time to onset of PM				0.136
Synchronous	96 (54.2)	69 (51.1)	27 (64.3)	
Differentiation grade				0.037
Poor	35 (19.8)	31 (23.0)	4 (9.6)	
*Operative characteristics*				
Peritoneal Cancer Index [median (range)]	10 (0–30)	9 (0–28)	11 (2–30)	0.011
CC score				0.007
CC0	166 (93.8)	130 (96.3)	36 (85.7)	
*Postoperative characteristics*				
Complication grade				0.839
CD1–2	43 (24.3)	32 (23.7)	8 (21.6)	
CD3–4	30 (16.9)	22 (18.5)	4 (10.8)	
Postoperative death (CD5)	3 (1.8)	3 (2.2)	–	
Hospital stay, days (range)	15.7 (2–80)	16.1 (2–80)	14.3 (8–59)	0.355
ICU stay, days (range)	2.6 (1–69)	2.8 (1–69)	1.8 (1–4)	0.321

Data are expressed as *n* (%) unless otherwise specified

*CRS* cytoreductive surgery, *HIPEC* hyperthermic intraperitoneal metastases, *SD* standard deviation, *ASA* American Society of Anesthesiologists Physical Status Classification System, *PM* peritoneal metastases, *CC* completeness of cytoreduction, *CD* Clavien–Dindo classification, *ICU* intensive care unit

T-test for different subgroups; Chi-square or Fisher's exact test

### Patients Not Eligible for Cytoreductive Surgery/Hyperthermic Intraperitoneal Chemotherapy

Patient characteristics and the reasons for not pursuing CRS/HIPEC are summarized in electronic supplementary Table 2. The main reasons for not performing CRS/HIPEC were PCI score >20 (28/58, 48%), systemic metastases (12/58, 21%), comorbidity (9/58, 16%), and irresectability (7/58, 12%). Sixteen of the 58 patients (28%) who did not undergo CRS/HIPEC were diagnosed with ascites on the preoperative CT. Ascites was more often present in patients with a PCI score >20 (11/28, 39%).

### Follow-Up and Predictors for Survival

**Fig. 2:**
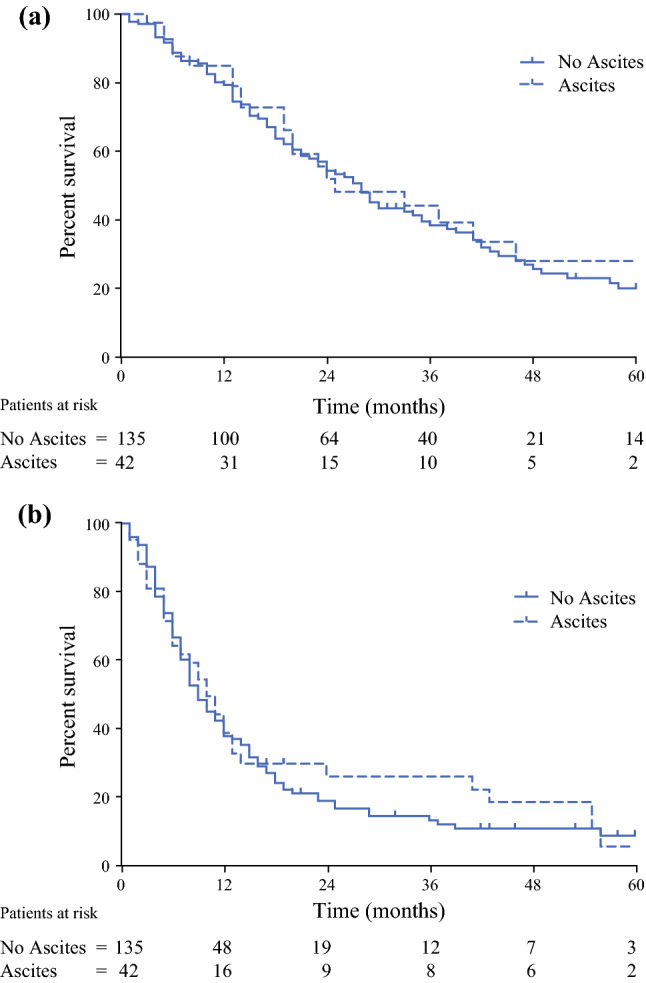
(**a**) Overall survival in patients with and without ascites who underwent CRS/HIPEC (log-rank [Mantel–Cox] test *p* = 0.26). (**b**) Disease-free survival in patients with and without ascites who underwent CRS/HIPEC (log-rank [Mantel–Cox] test *p* = 0.7)

The median follow-up of all patients was 15 months (range 0.2–105) and the median OS after CRS/HIPEC was 27 months (95% confidence interval [CI] 22.5–31.5). Survival was significantly worse for patients not eligible for surgery, with a median OS of 4 months (95% CI 3–5). The presence of ascites did not influence OS after CRS/HIPEC, as patients with ascites had a median OS of 25 months (95% CI 9.4–40.6) compared with 27 months (95% CI 22.4–31.6) for patients without ascites (*p* = 0.54) [Fig. 2a].

During follow-up, 123 of 177 CRS/HIPEC patients (69.5%) were diagnosed with recurrent disease; the median DFS was 9 months. There was no significant difference in DFS between patients with and without ascites (10 months [95% CI 7.1–12.9] vs. 9 months [95% CI 7.2–10.8]; *p* = 0.81) [Fig. 2b]. Furthermore, there was no significant differences in recurrence pattern in either the lung (13/123, 10.6%), liver (49/123, 39.8%), or peritoneal (61/123, 49.5%) between patients with and without ascites (*p* = 0.775). The median and 3- and 5-year OS and DFS for both groups are presented in Table 2.

**Table 2:** Overall and disease-free survival in patients undergoing CRS/HIPEC [*n* = 177]

	Total [*n* = 177]	No ascites [*n* = 135]	Ascites [*n* = 42]	*p*-Value
*OS*				
Median (95% CI) 3-year (%) 5-year (%)	27.0 (22.5–31.5) 39.2 20.9	27.0 (22.4–31.6) 37.9 19.8	25.0 (9.4–40.6) 44.1 28.0	0.536
*DFS*				
Median (95% CI) 3-year (%) 5-year (%)	9 (7.4–10.6) 14.5 7.9	9 (7.2–10.8) 13.5 9.4	10 (7.1–12.9) 18.9 4.7	0.814

*CRS* cytoreductive surgery, *HIPEC* hyperthermic intraperitoneal metastases, *OS* overall survival, *DFS* disease-free survival, *CI* confidence interval

Multivariate Cox regression analysis identified pT stage (hazard ratio [HR] 1.2, 95% CI 1.0–1.3), pN stage (HR 2.0, 95% CI 1.4–3.0), signet ring cell histology (HR 1.7, 95% CI 1.2–2.6), PCI score ≥7 (HR 1.6, 95% CI 1.1–2.4), and incomplete cytoreduction (HR 1.9, 95% CI 1.3–2.7) as independent prognostic factors for worse survival in CRPM patients who underwent CRS/HIPEC. Unadjusted and adjusted HRs are shown in Table 3.

**Table 3:** Univariate and multivariate analysis for overall survival in patients undergoing CRS/HIPEC [*n* = 177]

Factors^a^	Unadjusted HR (95% CI)		*p*-Value^b^	Adjusted HR^†^ (95% CI)		*p*-Value^c^
*Patient characteristics*						
Female sex	1.1	(0.7–1.5)	0.73			
ASA 3^a^	1.1	(0.9–1.3)	0.55			
*Preoperative characteristics*						
Ascites on CT^a^	0.9	(0.5–1.4)	0.54			
*Tumor characteristics*						
pT stage 4^a^	1.2	(1.0–1.3)	0.03	1.2	(1.0–1.3)	0.04
pN stage 2^a^	2.2	(1.5–3.2)	< 0.01	2.0	(1.4–3.0)	< 0.01
Differentiation-poor	1.5	(1.2–1.8)	< 0.01	1.2	(0.9–2.6)	0.18
Histology signet ring cell carcinoma	2.0	(1.4–2.7)	< 0.01	1.7	(1.2–2.6)	< 0.01
*Operative characteristics*						
PCI score ≥7	1.5	(1.2–1.9)	< 0.01	1.6	(1.1–2.4)	< 0.01
>CC0^a^	2.0	(1.4–2.7)	< 0.01	1.9	(1.3–2.7)	< 0.01

Factors analyzed in univariate analyses that were not significant included age, comorbidity, time to onset of peritoneal metastases, HIPEC regimen, presence of ovarian metastases, and complication grade. Other variables analyzed but not significant were Clavien–Dindo score, HIPEC chemotype oxaliplatin and mitomycin C, and comorbidity such as smoking, hypertension, diabetes, and cardiovascular disease

*CRS* cytoreductive surgery, *HIPEC* hyperthermic intraperitoneal metastases, *HR* hazard ratio, *CI* confidence interval, *ASA* American Society of Anesthesiologists Physical Status Classification System, *CT* computed tomography, *PCI* Peritoneal Cancer Index, *CC* completeness of cytoreduction

^a^The HR for this variable was compared with patients who were negative for this variable

^b^Value of the log-rank test

^c^*P*-value of the remaining significant independent variables after multivariate Cox regression analysis

## Discussion

In the present study, we assessed the predictive value of ascites on preoperative CT imaging to improve the selection of CRPM patients for CRS/HIPEC. We found that ascites was not associated with worse survival in patients who underwent CRS/HIPEC. Our study showed that ascites is present in 24% of patients eligible for CRS/HIPEC, which is similar to the incidence of ascites in patients who did not undergo CRS/HIPEC for peritoneal metastases. Ascites was however associated with higher PCI and a lower rate of complete cytoreduction.

CRS/HIPEC has improved survival outcomes for patients with CRPM but is associated with significant morbidity and mortality. Based on previous studies, high PCI score, incomplete cytoreduction and the histological subtype of the primary tumor have been identified as important predictors of early recurrence after CRS/HIPEC.^6^

A subgroup of patients experienced early recurrence after HIPEC despite favorable PCI and resectable lesions. Our study shows that the presence of ascites on imaging should not be decisive in patient selection for CRS/HIPEC; thus, additional selection criteria are required to identify this subgroup. Sampling and cytological evaluation of intra-abdominal fluid during exploratory laparoscopy may be of prognostic value, which should be explored in future research.

Leimkühler et al.^14^ showed that the addition of diagnostic laparoscopy to CT imaging leads to a clinically relevant but statistically insignificant reduction in the rate of open/close procedures and recommended adding diagnostic laparoscopy to CT imaging when the PCI score exceeds 10. Since our study shows that ascites is associated with a higher PCI score, we are in favor of additional diagnostic laparoscopy to CT imaging to mitigate the risk of an open/close procedure in patients with ascites. This is currently not standard of care, as, in our prospective cohort, only 48% (20/42) of patients with ascites underwent a diagnostic laparoscopy prior to CRS/HIPEC. Other imaging modalities, such as diffusion-weighted magnetic resonance imaging (MRI), could also contribute to a more reliable PCI prior to surgery. This is currently under investigation in the DISCO trial.^15^

Furthermore, patients with peritoneal metastases and ascites might respond differently to neoadjuvant chemotherapy before CRS/HIPEC. The CAIRO6 trial investigated, in a randomized fashion, whether the addition of perioperative systemic therapy to CRS/HIPEC improved oncological outcome.^16^ This study may show that specific patient groups, including patients with ascites, may benefit more or less from neoadjuvant chemotherapy. As such, ascites on CT imaging might have clinical consequences in the future treatment of patients with CRPM.

Patient series in ovarian and gastric cancer show that ascites was associated with advanced disease stage.^17,18^ In our cohort, the presence of ascites was also associated with an increased PCI. However, CRC patients with ascites who underwent CRS/HIPEC did not have worse DFS or OS compared with patients without ascites. Other factors, including primary tumor characteristics (pT stage, pN stage, signet ring cell histology), as well as PCI and CC, were predictive of OS, as previously identified.^6^

The present findings show that ascites was not associated with worse survival after CRS/HIPEC. This suggests that in some patients, the observed ascites could be reactive fluid rather than malignant ascites containing tumor cells. Other causes, such as infection and cardiac or hepatic disease, could explain the presence of ascites, but are probably less prevalent in our patient cohort. The pathophysiology of malignant ascites is different from hepatic ascites. Ascites formation from cirrhosis is theorized to be via peripheral arterial vasodilation, while the pathophysiology of malignant ascites is thought to be a combination of altered vascular permeability and obstructed lymphatic drainage.^19,20^ Positive cancer cell cytology of peritoneal fluid has been identified as an independent negative prognostic factor in patients with CRPM undergoing CRS/HIPEC.^21^

## Conclusion

We found no correlation between the presence of ascites on preoperative imaging and survival outcome after CRS/HIPEC for CRPM. We therefore suggest that excluding patients from CRS/HIPEC based merely on the presence of ascites is not advisable. Considering that ascites was associated with higher PCI, the present findings may be helpful in multidisciplinary team discussions and could be used to counsel patients towards diagnostic laparoscopy to reduce a futile laparotomy because of excessive peritoneal disease.

## Supplementary Information

Below is the link to the electronic supplementary material.

Supplementary file1 (DOCX 16 kb)
