# Supplementary material for: In Patients Undergoing CRS/HIPEC for Colorectal Adenocarcinoma with Peritoneal Metastases, Presence of Ascites on Computed Tomography Imaging is not a Prognostic Marker for Survival
Source: Ann Surg Oncol. 2022 Apr 16;29(8):5256–62. doi: 10.1245/s10434-022-11718-7 (PMC9246798; doi:10.1245/s10434-022-11718-7)
Supplement: Supplementary file 1 — Supplementary file1 (DOCX 16 kb) [file 10434_2022_11718_MOESM1_ESM.docx]

Supplementary files

| **Supplementary Table 1.**  **The association between ascites distribution, Peritoneal Cancer Index and complete cytoreduction.** | | | | |
| --- | --- | --- | --- | --- |
|  | **All patients** | | | |
|  | N=42 |  |  |  |
| Ascites distribution score | PCI (median, range) | CC0 [n[%]] | CC1-2 [n[%]] | P-value |
|  |  |  |  | 0.944 |
| *Low* | 12 (4-28) | 22 (84.6) | 4 (15.4) |  |
| *Medium* | 9 (4-14) | 5 (100) | 0 (0) |  |
| *High* | 14 (2-30) | 9 (81.8) | 2 (18.2) |  |
| Values in parentheses are percentages unless indicate otherwise  PCI, Peritoneal Cancer Index; CC, Completeness of Cytoreduction; CC0 complete, CC1-2 incomplete | | | | |

| **Supplementary Table 2.**  **Baseline characteristics patients not eligible for CRS-HIPEC** | | |
| --- | --- | --- |
|  | **All patients** | |
| Total patients | n=58 |  |
| Patient characteristics |  |  |
| Gender |  |  |
| *Male [n(%)]* | 24 (41.4) |  |
| Age [year; mean ± sd] | 65.4 ± 11.3 |  |
| ASA |  |  |
| *ASA ≥3 [n(%)]* | 9 (15.5) |  |
| Race |  |  |
| *White* | 58 (100) |  |
| Tumor characteristics |  |  |
| pT stage |  |  |
| *4 [n(%)]* | 13 (22.4) |  |
| pN stage |  |  |
| *2 [n(%)]* | 10 (17.2.) |  |
| Time to onset of PM |  |  |
| *Synchronous [n(%)]* | 30 (51.7) |  |
| Ascites on CT | 16 (27.6) |  |
| Reasons not to perform CRS-HIPEC |  |  |
| *Systemic metastases [n(%)]* | 12 (20.7) |  |
| *Irresectability [n(%)]* | 7 (12.1) |  |
| *High PCI [n(%)]* | 28 (48.3) |  |
| *Comorbidity/Performance status [n(%)]* | 9 (15.5) |  |
| *Age [n(%)]* | 1 (1.7) |  |
| *Shared decision making [n(%)]* | 1 (1.7) |  |
| Values in parentheses are percentages unless indicate otherwise ASA, American Society of Anesthesiology Physical Status Classification System; PCI, Peritoneal Cancer Index | | |
